# New Mitochondrial Genomes of Ithonidae (Neuroptera) and Higher Phylogenetic Implications

**DOI:** 10.3390/insects15120933

**Published:** 2024-11-27

**Authors:** Ruyue Zhang, Yunlan Jiang, Mina Zhong, Shutong Wang, Yuyu Wang

**Affiliations:** 1 College of Plant Protection, Hebei Agricultural University, Baoding 071001, China; ruyue_zhang@126.com (R.Z.); yunlan_jiang@163.com (Y.J.); bdstwang@163.com (S.W.); 2 Forest Pest Control and Quarantine Station of Xining City, Xining 810008, China; shezmn@126.com

**Keywords:** mitochondrial genome, Ithonidae, phylogenetic relationships

## Abstract

Ithonidae (moth lacewings) are an enigmatic, small family of the insect order Neuroptera (lacewings). Its phylogenetic position within Neuroptera and internal subfamily relationships remain unresolved. In this study, the complete mitochondrial genome (mitogenome) of *Ithone fulva* Tillyard, 1916 representing the first mitogenome of Ithoninae, as well as the complete mitogenome of *Rapisma gaoligongensis* Liu, Li and Yang, 2018, were newly reported. Molecular phylogenetic trees recovered Ithonidae as the sister group to Chrysopidae + Hemerobiidae. Ithoninae was demonstrated to be the sister group to Polystoechotinae + Rapismatinae across all topologies. Divergence time estimation revealed that Ithonidae originated during the Mid-Triassic. Ithoninae diverged from Polystoechotinae and Rapismatinae in the Late Triassic, while Polystoechotinae diverged from Rapismatinae in the Middle Jurassic. Moreover, more comprehensive samplings, as well as whole genome data, are needed to reconstruct a comprehensive framework of the phylogeny of Ithonidae, as well as Neuropteida.

## Simple Summary

Ithonidae (moth lacewings) are an enigmatic, small family belonging to Neuroptera (lacewings). In this study, two complete mitochondrial genomes (mitogenomes) of moth lacewings were sequenced and analyzed for the first time. Comparative analyses of the mitogenomes of Ithonidae were conducted, such as codon usage, nucleotide substitution rates and secondary structure predictions of RNAs. Molecular phylogenetic trees recovered that Ithonidae was a monophyletic group, and is the sister-group to the clade of Chrysopidae + Hemerobiidae. Ithoninae was recovered as the sister group to Polystoechotinae + Rapismatinae. In addition, divergence time estimation indicated that Ithonidae started to diversify in the Late Triassic. Polystoechotinae diverged from Rapismatinae in the Middle Jurassic.

## 1. Introduction

Ithonidae (moth lacewings) are a small and ancient group belonging to Neuroptera, which are distributed throughout Asia, Australia, Oceania, North and South America [1]. Adults are usually medium to large in size, with stout antennae, a broad and short head which is retracted under the pronotum, a cylindrical abdomen, and broad wings with pectinate veinlets on both forewings and hindwings [2]. Larvae are usually robust, grub-like (scarabaeiform) with stout mandibles, presenting an unusual subterranean lifestyle, feeding on root exudates [2,3,4,5]. Notable sexual dimorphism has been documented and observed in certain species of Ithonidae [6,7].

The phylogenetic placement and internal phylogenetic relationships of Ithonidae have remained unresolved. Ithonidae sensu stricto has been considered a separate group from Polystoechotidae and Rapismatidae for a long time [8,9,10,11]. However, Ithonidae s. s. were demonstrated to be paraphyletic, with the genus *Oliarces* being assigned to Polystoechotidae based on four genes (*CAD*, *18S rRNA*, *COX1* and *16S rRNA*) and morphological data [12]. Moreover, Ithonidae s. s. and Rapismatidae were distinguished from each other based on eight plesiomorphic characteristics [13]. The validity of Rapismatidae has been questioned [14]. Polystoechotidae and Rapismatidae were included in Ithonidae sensu lato based on 2 nuclear genes (*CAD*, *18S rRNA*), 1 mitochondrial gene (*16S rRNA*) and 23 morphological characters, as well as fossil evidences [2], which is now widely accepted [15,16,17,18,19,20,21]. According to the high-level classification by Neuropterida Species of the World, Ithonidae s. l. are divided into three subfamilies, i.e., Ithoninae (ca. 12 species), Polystoechotinae (ca. four species) and Rapismatinae (ca. 28 species) [22]. To date, the phylogenetic relationships of these groups are still uncertain. Ithonidae s. l. have been proposed to be a sister group to Nymphidae based on Anchored Hybrid Enrichment (AHE) data [16,20,21], to Chrysopidae based on mitogenome data [6,17], and to Myrmeleontiformia based on both mitogenome [17,19] and transcriptome data [23]. The phylogenetic placement of Ithonidae s. l. has still been under debate.

Currently, there are only three complete mitogenomes (*Oliarces clara* Banks, 1908, *Polystoechotes punctata* (Fabricius, 1793) and *Rapisma zayuanum* C.-k. Yang, 1993) and one incomplete mitogenome (*Fontecilla graphicus* Navás, 1932) of Ithonidae available in GenBank (http://www.ncbi.nlm.nih.gov (accessed on 11 August 2024)), with only one representative of *Rapisma*, and no representative of Ithoninae. In this study, the complete mitogenomes of *Ithone fulva* Tillyard, 1916 [24] (Neuroptera: Ithonidae: Ithoninae) and *Rapisma gaoligongensis* Liu, Li and Yang, 2018 [25] (Neuroptera: Ithonidae: Rapismatinae) were newly sequenced. Comparative analyses of the mitogenomes of Ithonidae, such as codon usages, nucleotide substitution rates and secondary structures of RNAs, were conducted. In addition, the phylogeny and estimation of divergence times of Ithonidae, as well as Neuropterida, were presented based on the mitogenome data.

## 2. Materials and Methods

### 2.1. Sampling, DNA Extraction and Sequencing

The specimen of *I. fulva* (GenBank accession no. OR506419) was collected by J. Skevington at Rainbow Beach, Queensland, Australia. The specimen of *R. gaoligongensis* (GenBank accession no. OR506418) was collected by Yuchen Zheng and Jiamin Zhang at Longyang District, Baoshan City, Yunnan Province, China. Vouchers of all specimens sequenced are deposited in 95% ethanol at −20 °C atthe Hebei Agricultural University (Baoding, China). Total genomic DNA was extracted using the DNeasy Blood and Tissue kit (QIAGEN, Hilden, Germany) from thoracic muscles. Illumina paired-end library (with 350 bp insert size, PE150) were sequenced on the Illumina NovaSeq 6000 platform by Novogene Bioinformatics Co., Ltd. (Beijing, China).

### 2.2. Genome Assembly and Annotation

Raw reads from the high-throughput sequencing were checked using FastQC v0.11.9 [26] with the default commands. The low-quality reads and adapters were filtered using Trimmomatic v0.32 [27]. The mitogenome of *I. fulva* (EU839734) was assembled by Novoplasty v2.7 [28] using the *COX1* sequence from the same species, while the mitogenome of *R. gaoligongensis* used the *COX1* sequence from the relative species (*R. zayuanum*, NC_023363) as baits. The mitogenomes were annotated using the MITOS Web Server [29] and then checked by manual proofreading. The PE reads were mapped to the final mitogenome assembly using BWA v0.7.5 [30] and SAMtools v1.2 [31]. The mitogenome maps were drawn with the OGDRAW web server [32]. The base composition and codon usage were analyzed using MEGA v7.0 [33]. The nucleotide diversity (Pi) and non-synonymous/synonymous substitution ratios (Ka/Ks) were calculated using DnaSP v6.12.0391 [34]. The relative synonymous codon usage (RSCU) of protein-coding genes (PCGs) was analyzed using MEGA v7.0 [33]. The secondary structures of tRNAs were inferred using the MITOS Web Server [29]. The secondary structures of rRNAs (*rrnL* and *rrnS*) were inferred using RNA Structure v6.5 [35].

### 2.3. Phylogenetic Analysis

In this study, the ingroup comprises 35 species of Neuroptera, including two newly sequenced mitogenomes of Ithonidae. The outgroup comprises six species of Megaloptera and four species of Raphidioptera (Table S1). The PCGs were aligned using MAFFT v7.182 [34] with ‘L-INS-I’. Each rRNA gene was aligned by MAFFT v7.182 [36] with ‘G-INS-I’. The PCGR dataset (13 PCGs and 2 rRNAs) was concatenated using FASconCAT v1.0 [37]. Ambiguously aligned positions of PCGs and rRNAs were excluded using Gblocks v0.91b [38]. The PCGR_Gb dataset containing 13 PCGs and 2 rRNAs masked by Gblocks v0.91b [38] was concatenated using FASconCAT v1.0 [37]. In addition, the 39taxa_PCGR dataset was concatenated, excluding Dilaridae and Raphidioptera, to reduce the potential effect of long-branch attraction on the topology. There are many studies demonstrating that applying the site-heterogeneous model (CAT-GTR) could reduce sequence heterogeneity and produce more plausible phylogenetic inferences [39,40,41,42]. Therefore, Bayesian inference (BI) analyses under the site-heterogeneous model (CAT-GTR) were used to reconstruct the phylogeny with PhyloBayes v3.3 [43] based on the PCGR, PCGR_Gb and 39taxa_PCGR datasets. Two independent Markov Chain Monte Carlo (MCMC) chains ran at the same time, until converged satisfactorily (maxdiff < 0.1, the minimum effective size > 200) with a burn-in of 25%. The trees were visualized using FigTree v1.4.4 [44].

### 2.4. Divergence Time Estimation

Divergence times for Ithonidae, as well as Neuropterida, were estimated using MCMCTree v4.9e [45]. The PCGR dataset and the corresponding topology obtained under the CAT-GTR model were selected as the input files. The age of the oldest fossil was used as a minimum age constraint in this study [46]. In addition, there were 20 minimum age constraints based on fossils that corresponded to either the crown or stem members of each of the families of Neuropterida. The details of each fossil, including age, taxonomic status and placement in the topology are summarized in Table 1. Additionally, the maximum age (320 Mya) at the root was used in lacewing dating estimation [47]. The independent rate clock model (clock = 2) was used for further calculation. Two independent MCMC chains were conducted for five million generations with 25% burn-in. The convergence of the MCMC chains was evaluated using Tracer v1.7.2 [48], ensuring that the ESS values exceeded 200.

## 3. Results and Discussion

### 3.1. Subsection Genome Organization and Base Composition

**Figure 1:**
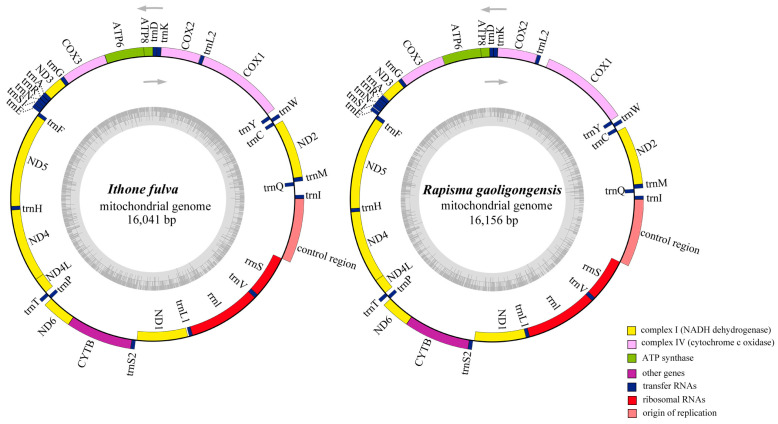
Mitochondrial maps of *Ithone fulva* and *Rapisma gaoligongensis*. Genes outside the map are transcribed counterclockwise, whereas those inside are transcribed clockwise.

The newly sequenced complete mitogenomes of *I. fulva* and *R. gaoligongensis* were traditional double-strain circular molecules of 16,041 and 16,156 bp in length, respectively (Figure 1, Tables S2 and S3). The sequencing depth and coverage map of the mitogenomes of *I. fulva* (maximal depth = 6262 X, minimal depth = 98 X and average depth = 2083.35 X) and *R. gaoligongensis* (maximal depth = 5806 X, minimal depth = 82 X and average depth = 2169.23 X) are presented in Figure S1. The gene composition and structural features of these two mitogenomes were consistent with that of other published Ithonidae species [6,19,67].

**Figure 2:**
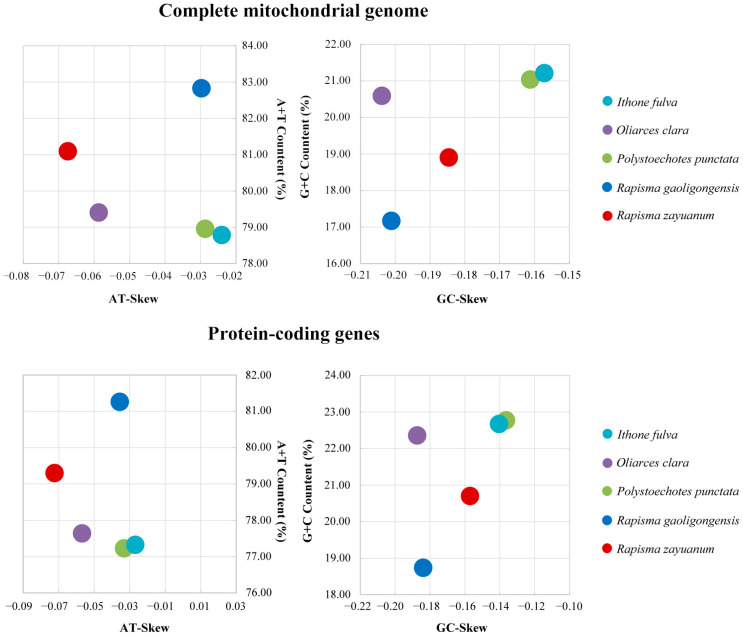
AT% vs. AT-Skew and GC% vs. GC-Skew of the whole complete mitochondrial genome, as well as the protein-coding genes, of Ithonidae.

The complete mitogenomes of Ithonidae ranged from 15,984 bp (*R. zayuanum*) to 16,156 bp (*R. gaoligongensis*) in length (Table S4). The content of A + T ranged from 78.79% to 82.83% (Figure 2, Table S4). The length of PCGs ranged from 11,115 bp of *I. fulva* to 11,173 bp of *O. clara* (Table S5). The A + T content of the PCGs ranged from 77.23% to 81.26%. The AT-skew and GC-skew of all complete mitogenomes as well as the PCGs were negative (Figure 2).

### 3.2. Protein-Coding Genes and Codon Usage

**Figure 3:**
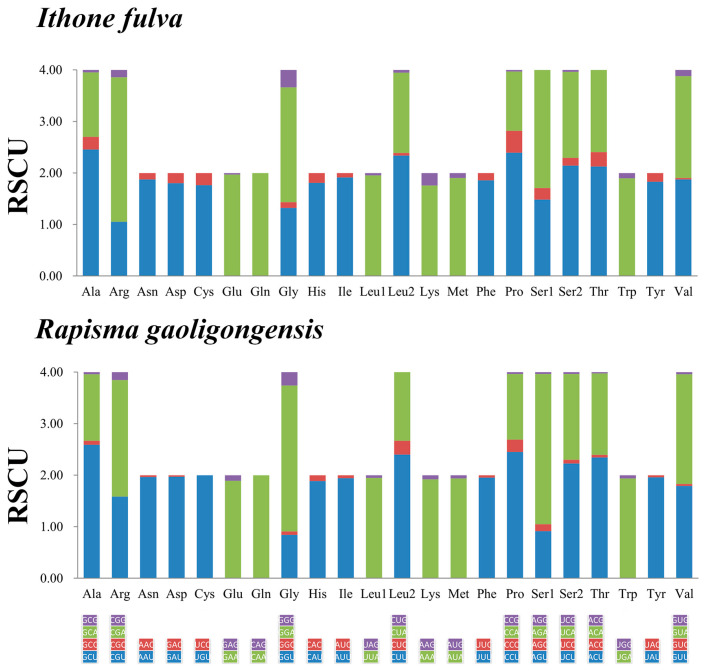
Relative synonymous codon usages (RSCUs) of protein-coding genes in the complete mitochondrial genome of *Ithone fulva* and *Rapisma gaoligongensis*. The RSCU values are color-coded based on the codons below the amino acid labels.

The relative synonymous codon usages (RSCUs) of PCGs of the mitogenomes of *I. fulva* and *R. gaoligongensis* are shown in Figure 3 and Tables S6 and S7. The highest value of RSCU in *R. gaoligongensis* and *I. fulva* were 2.92 of AGA and 2.81 of CGA, respectively. The TTA (Leu1), ATT (Ile), TTT (Phe) and ATA (Met) were the most frequently used codons, indicating the preference of nucleotide composition A and T (Table S8).

Generally, the start and stop codons of PCGs of complete mitogenomes of Ithonidae were relatively similar. Most PCGs started with the typical initiation codon ATN. Specifically, *COX1* used CGA in *O. clara* and TCG in *P. punctata* as the start codon, while *ND1* used TTG as the start codon in *O. clara*, *R. gaoligongensis* and *R. zayuanum*, respectively. Regarding stop codons, *ATP6*, *ATP8*, *ND4L* and *ND6* consistently used TAA, while *ND1* terminated with TAG in all species. Some PCGs terminated with T-tRNA (*ND2*, *ND3*, *ND4 ND5 COX1*, *COX2* and *CYTB*) or TA-tRNA (*COX3*) in some species of Ithonidae, which is universal in insects [68,69,70,71] (Table S9).

**Figure 4:**
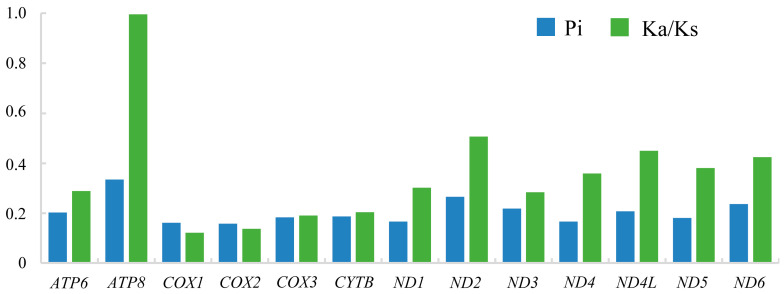
The nucleotide diversity (Pi) and non-synonymous (Ka) to synonymous (Ks) substitution rates of 13 protein-coding genes of the complete mitochondrial genome of Ithonidae.

The Pi and the Ka/Ks rate of 13 PCGs of the complete mitogenomes in Ithonidae are shown in Figure 4. The Pi of 13 PCGs ranged from 0.158 (*COX2*) to 0.334 (*ATP8*). The Ka/Ks rate of 13 PCGs ranged from 0.112 for *COX1* to 0.996 for *ATP8*. Notably, the Ka/Ks of all 13 PCGs were lower than 1, which indicated that all PCGs of the five complete mitogenomes of Ithonidae were under purifying selection [72,73].

### 3.3. Transfer RNAs and Ribosomal RNAs

**Figure 5:**
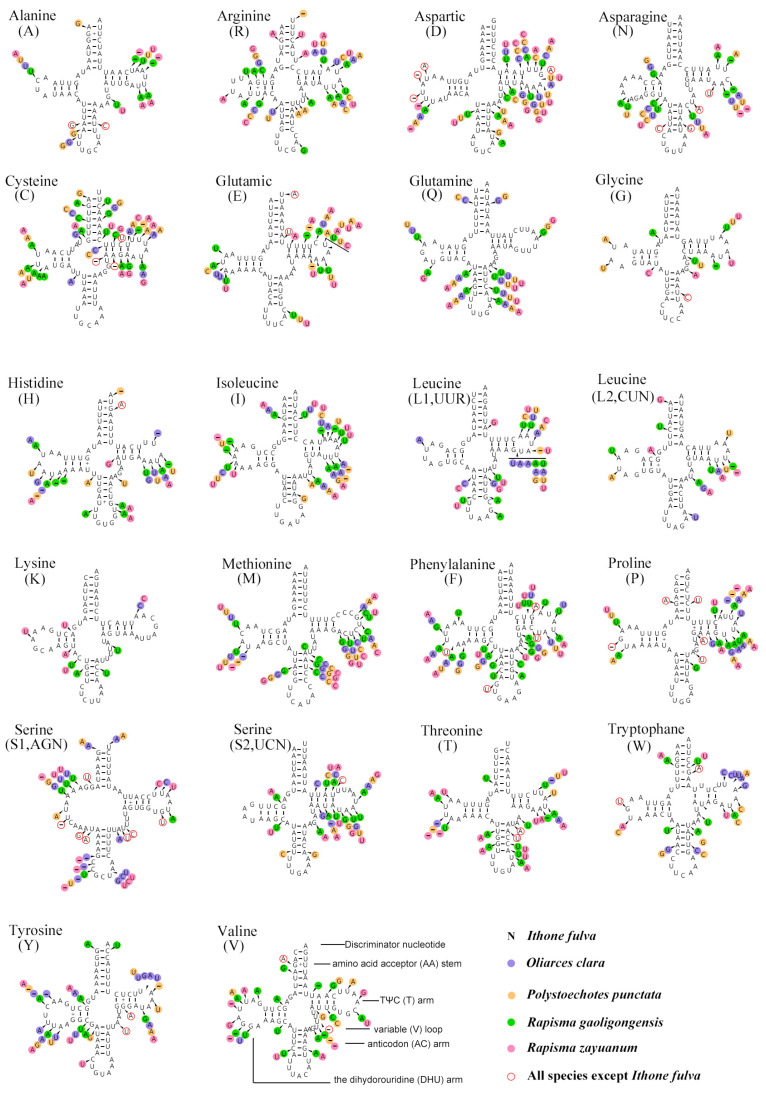
Inferred secondary structures of 22 tRNAs of the complete mitochondrial genome of Ithonidae.

The 22 tRNAs of the five complete mitogenomes of Ithonidae were typical, including all 20 types of amino acids (Figure 5). Almost all tRNAs could be folded into the classical cloverleaf-like structure with the exception of *tRNA*^Ser (AGN)^, whose dihydrouridine (DHU) arm just formed a loop. The gene with the highest rate of variable positions was *tRNA*^Cys^ (35.71%), while the gene with the lowest rate of variable positions was t*RNA*^Lys^ (9.72%).

**Figure 6:**
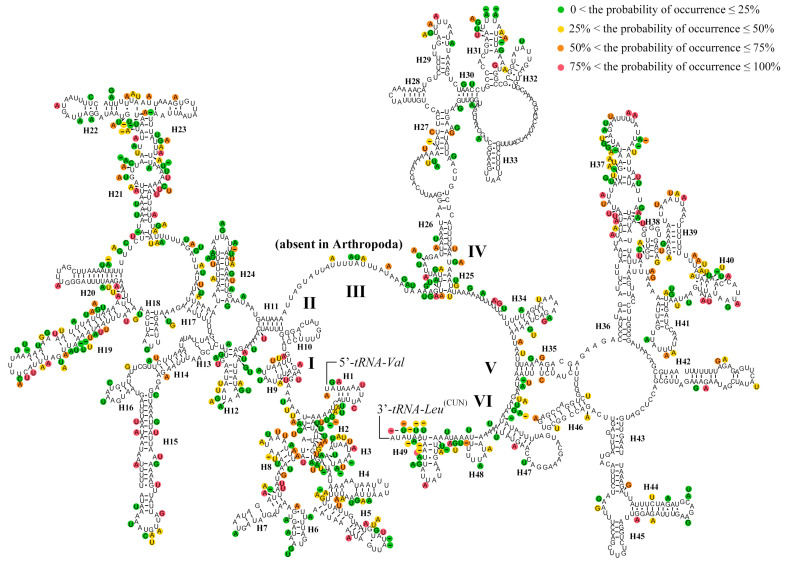
Inferred secondary structures of *rrnL* of the complete mitochondrial genome of Ithonidae.

**Figure 7:**
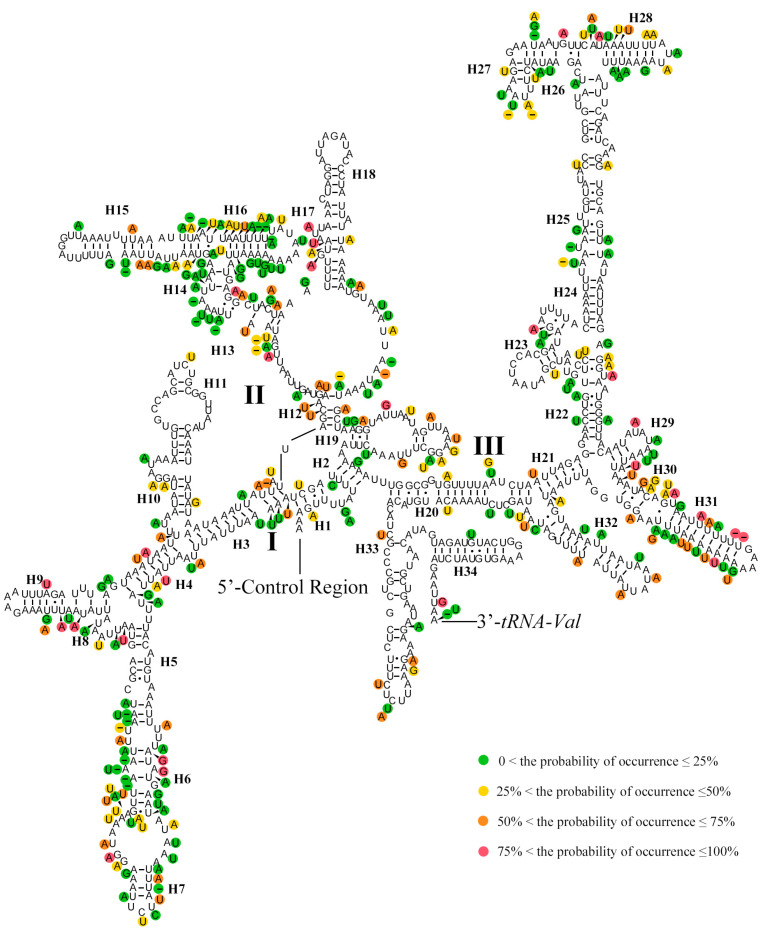
Inferred secondary structures of *rrnS* of the complete mitochondrial genome of Ithonidae.

The *rrnL* was located between *tRNA*^Leu (CUN)^ and *tRNA*^Val^ ranging from 1316 bp to 1327 bp long (Table S10). The A + T content of *rrnL* ranged from 81.50% to 83.59%. The AT-skew of the *rrnL* ranged from 0.00 to 0.07, while the GC-skew ranged from 0.26 to 0.34. The *rrnS* was located between *tRNA*^Val^ and the control region (CR) ranging from 777 bp to 795 bp long. Both the AT-skew and GC-skew values of the *rrnS* were positive. The predicted secondary structures of *rrnL* of the mitogenomes in Ithonidae consisted of five canonical domains (I–II, IV–VI) and 49 helices with domain III absent (Figure 6), which is typical in arthropods [74]. The rate of mutation was higher in domain I and II, and lower in domains IV and VI. The predicted secondary structure of *rrnS* of the mitogenome in Ithonidae consisted of three domains and 34 helices (Figure 7). The mutation rate of each position ranged mostly from 0 to 25% in the predicted secondary structure of *rrnL* and *rrnS* with *I. fulva* as reference.

### 3.4. Control Region

**Figure 8:**
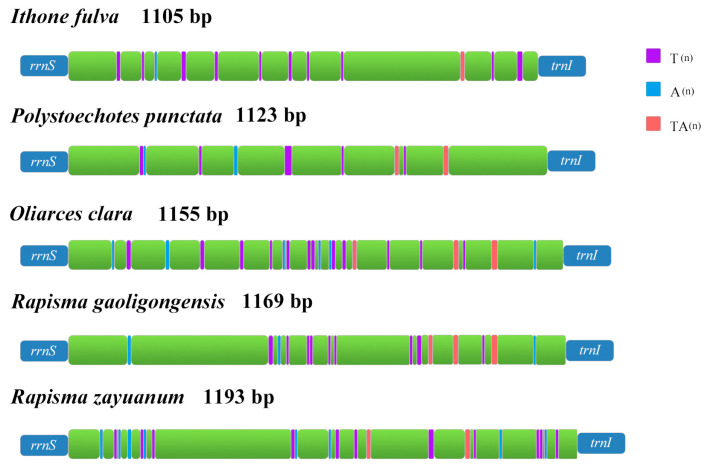
Structures of the control region of the complete mitochondrial genome of Ithonidae.

The CR in *I. fulva* and *R. gaoligongensis* was located in *tRNA*^Val^ and *tRNA*^Ile^. In the complete mitogenome of Ithonidae, the length of the CR ranged from 1105 to 1193 bp (Table S11). The A + T content of CR ranged from 86.43% to 94.89%. The AT-skew ranged from −0.10 to 0.01, while the GC-skew ranged from −0.57 to −0.16. Many microsatellite-like sequences such as (TA)n were present in the CR of the five complete mitogenomes of Ithonidae (Figure 8), which may facilitate the completion of DNA replication and transcription and serve as useful markers for geographical studies [68,75].

### 3.5. Phylogenetic Analyses

**Figure 9:**
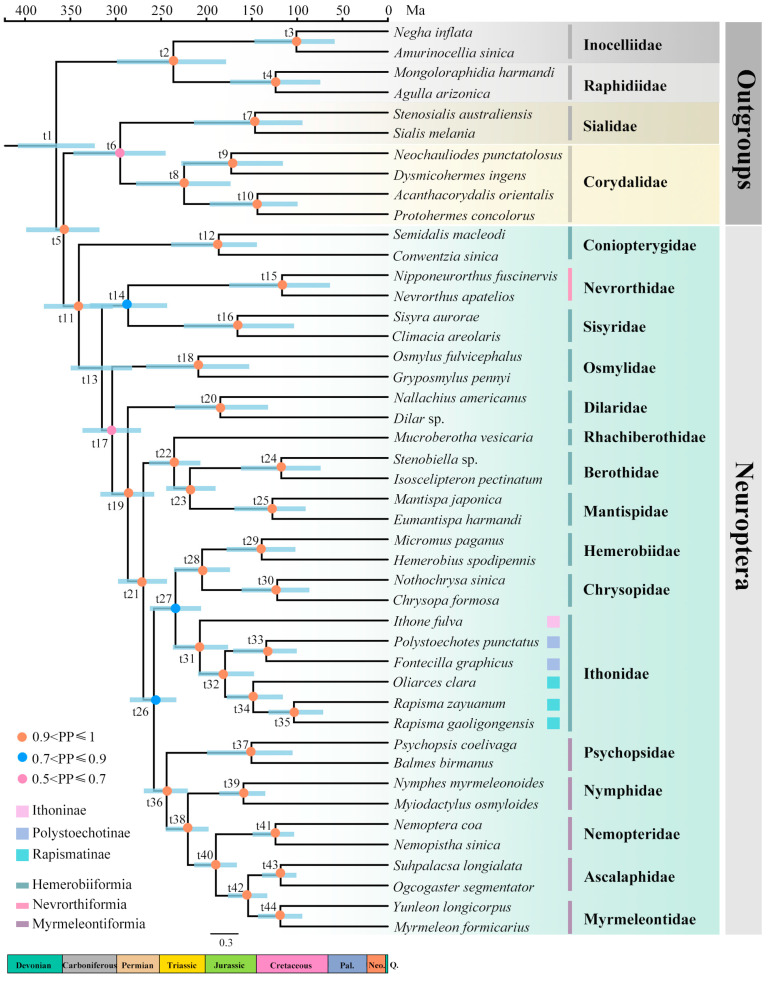
Phylogenetic relationships and divergence time estimation of all families of Neuropterida. Node numbers refer to values of posterior probabilities (PP) and estimated ages presented in Table 2. Yellow circles represent nodes with PP values above 0.9. Blue circles represent nodes with PP values between 0.7 and 0.9, and pink circles represent PP values between 0.5 and 0.7.

There are three phylogenetic trees reconstructed based on BI analyses under the site-heterogeneous model with consistent topologies in this study (Figure 9, Figures S2 and S3). Neither the removal of ambiguously aligned positions nor the removal of Dilaridae and Raphidioptera had any effect on the topology.

The monophyly of Ithonidae was supported in all phylogenetic trees. Ithonidae was recovered as the sister group to the clade of Chrysopidae + Hemerobiidae by all phylogenetic analyses with low supports. Some studies formerly demonstrated that Ithonidae belonged to Hemerobiiformia, close to Hemerobiidae or Chrysopidae, based on molecular data [6,17,75,76,77,78,79] and the morphological characteristics of the larval head [11]. However, the sister-group relationship between Ithonidae and Myrmeleontiformia was recovered based on mitogenome [19] and transcriptome data [23] with limited samples, while the sister-group relationship between Ithonidae and Nymphidae was recovered based on AHE data [16]. There are only two additional species in the current study compared with the former study by Wang et al. (2017) [19]. However, the topologies were different even when the same methods were used, which indicated that more samplings as well as the whole genomic data would be needed for future exploration. Within Ithonidae, all trees consistently supported Ithoninae (*Ithone*) being the sister group to the remaining Ithonidae, as well as Polystoechotinae being the sister group to Rapismatinae. *Oliarces* was supported as the sister group to *Rapisma*, while *Polystoechotes* was recovered as the sister group to *Fontecilla* based on all phylogenetic trees.

Furthermore, the monophyly of Neuroptera and Coniopterygidae being the sister group to all the remaining families of Neuroptera were recovered, which is consistent with other cladistic analysis based on morphological [9,80,81,82,83] as well as molecular data [16,17,19,23,84,85]. The branch of Nevrorthidae + Sisyridae was recovered as the sister group to the rest of Neuroptera except Coniopterygidae, followed by Osmyloidae, which is in agreement with the results of analyses based on former mitogenome data [17,19]. Dilaridae was recovered as the sister group to all of the remaining families of Neuroptera with the exception of Coniopterygidae and Osmyloidea in all analyses, which is consistent with other cladistic analysis based on combined *COX1*, *CAD*, *18S rRNA*, *16S rRNA* and morphological data [12], AHE data [16], transcriptome [23] and mitogenome data [17,19]. Rhachiberothidae was consistently recovered as the sister group to Berothidae and Mantispidae, similar to the studies based on AHE [16] and transcriptome [23]. Chrysopidae was recovered as the sister group to Hemerobiidae in all analyses, which agrees with previous molecular studies using mitogenome data [18,19,68] but disagrees with studies based on AHE data [16] and transcriptome data [23]. Psychopsidae was recovered as the sister group to all of the remaining Myrmeleontiformia in all analyses, which is consistent with other studies based on mitogenome data [17,19,79]. Nymphidae was recovered as the sister group to the clade comprising Nemopteridae + (Myrmeleontidae + Ascalaphidae) in all analyses, which is consistent with recent molecular studies based on transcriptome [23] and mitogenome data [19].

On the basis of the above analyses, the incongruences phylogenies based on mitogenome and nuclear genome cannot be easily dismissed. The mitogenome is relatively independent from the nuclear genome and has its own mechanism of inheritance (matrilineal inheritance) and evolutionary dynamics [86,87]. This may be the potential reason for the conflicting phylogenetic topologies based on mitochondrial and nuclear genes. More comprehensive samplings, as well as the whole genome data, will be needed to explore the phylogeny of Ithonidae, and even Neuropterida, in the future.

### 3.6. Divergence Time Estimation

The divergence time estimates for Ithonidae, as well as Neuropterida, based on the tree using the PCGR dataset under the heterogeneous model (CAT-GTR), were shown in Figure 9 and Table 2. Ithonidae was inferred to be monophyletic and diverged from the clade comprising Chrysopidae and Hemerobiidae in Middle Triassic (mean = 234.12 Mya, HPD = 205.92–262.42). Ithoninae diverged from Polystoechotinae and Rapismatinae during Late Triassic (mean = 207.23 Mya, HPD = 176.14–237.05), which is older than the previously estimated time based on two nuclear genes (*CAD*, *18S rRNA*) and two mitochondrial genes (*COX1*, *16S rRNA*) (~177 Mya) [12], as well as AHE data (~156.71 Mya) [16]. Polystoechotinae diverged from Rapismatinae in the middle of Jurassic (mean = 179.46 Mya, HPD = 147.53–209.24). *Oliarces* diverged from *Rapisma* in the end of Jurassic (mean = 148.42 Mya, HPD = 115.70–177.42).

Megaloptera diverged from Neuroptera in Early Carboniferous (mean = 357.55 Mya, HPD = 317.95–398.92). Coniopterygidae was inferred to be the sister group to all of the remaining families of Neuroptera. Neuroptera started to diversify during Early Carboniferous (mean = 340.63 Mya, HPD = 303.26–379.21), older than the previous estimation during Permian based on AHE data (~267.38 Mya) [16], transcriptome data (~280.82 Mya) [23] as well as mitogenome data (~280.49 Mya) [19]. Osmylidae started to diversify during Late Triassic (mean = 208.93 Mya, HPD = 152.81–266.42), which is similar to the estimation based on transcriptome data (~206 Mya) [23]. Some neuropterid families (i.e., Coniopterygidae, Sisyridae, Dilaridae, Psychopsidae, Nymphidae) started to diversify during Jurassic. The other neuropterid families (i.e., Nevrorthidae, Berothidae, Mantispoidae, Hemerobiidae, Chrysopidae, Ascalaphidae, Nemopteridae, Myrmeleontidae) started to diversify during Cretaceous, which is consistent with the proposal by Wang et al. that Mesozoic was the ‘golden age’ of the lacewings [19].

The divergence time of some taxa in Neuropterida inferred using the mitogenome dataset was more ancient than that inferred using nuclear genes, e.g., Ithonidae. The rapid rate of evolution of mitochondrial DNA may be one of the reasons [88,89]. Additionally, the choice of fossil would also affect the assessment of divergent time [90,91]. The fossil calibrations used were almost the oldest fossils of the taxon at that time [92]. However, as fossils continue to be discovered, and the oldest fossil record is being updated [51,58]. The accuracy of the divergence time estimation based on these two types of molecular markers (mitochondrial and nuclear genes) could be evaluated using a consistent calibration scheme and the same set of samples in the future.

## 4. Conclusions

The complete mitogenome of *I. fulva* as the first representative of Ithoninae, as well as the complete mitogenome of *R. gaoligongensis* of the genus *Rapisma*, which has only one representative published to date, were reported in this study. It was found that the mitogenomes of Ithonidae had similar structural features and nucleotide composition. Ithonidae was recovered as monophyletic and being the sister group to the clade of Chrysopidae + Hemerobiidae. Molecular dating analyses suggested that Ithonidae originated during Mid-Triassic. Ithoninae diverged from Polystoechotinae and Rapismatinae in Late Triassic, while Polystoechotinae diverged from Rapismatinae during the middle of Jurassic. More comprehensive samplings, as well as whole genomic data, will be needed to better understand the phylogeny and divergence time estimation of Ithonidae, as well as Neuropteida, in the future.

## Figures and Tables

**Table 1:** Minimum age constraints used for the divergence times analysis in this study.

Node	Fossil Species	Family	Placement	Age (Ma)	References
t3	*Fibla erigena*	Inocelliidae	Crown Inocelliidae	41.0	Carpenter (1956) [49]
t4	*Raphidia baltica*	Raphidiidae	Crown Raphidiidae	34.0	Carpenter (1956) [49]
t5	*Permoberotha villosa*	Permoberothidae	Crown Neuroptera + Megaloptera	280.0	Tillyard (1932) [50]
t6	*Izyumochauliodes aristovi*	Corydalidae	Crown Megaloptera	205.7	Prokin et al. (2023) [51]
t12	*Juraconiopteryx zherichini*	Coniopterygidae	Crown Coniopterygidae	156.0	Meinander (1975) [52]
t15	*Palaeoneurorthus groehni*	Nevrorthidae	Crown Nevrorthidae	50.0	Wichard et al. (2010) [53]
t16	*Prosisyrina sukachevae*	Sisyridae	Crown Sisyridae	85.0	Perkovsky and Makarkin (2015) [54]
t17	*Lithosmylidia baronne*	Osmylidae	Stem Osmylidae	242.0	Lambkin (1988) [55]
t18	*Juraheterosmylus antiquatus*	Osmylidae	Crown Osmylidae	156.0	Wang et al. (2010) [56]
t20	*Cascadilar eocenicus*	Dilaridae	Crown Dilaridae	50.0	Engel (1999) [57]
t23	*Triassoberotha japonica*	Berothidae	Stem Berothidae	226.8	Khramov et al. (2023) [58]
t24	*Elektroberotha groehni*	Berothidae	Crown Berothidae	50.0	Makarkin and Ohl (2015) [59]
t27	*Guithone bethouxi*	Ithonidae	Stem Ithonidae	158.0	Zheng et al. (2016) [60]
t28	*Mesypochrysa minuta*	Chrysopidae	Stem Chrysopidae	165.0	Jepson et al. (2012) [61]
t36	*Triassopsychops superba*	Psychopsidae	Stem Psychopsidae	235.0	Tillyard (1922) [62]
t38	*Liminympha makarkini*	Nymphidae	Stem Nymphidae	172.0.	Ren and Engel (2007) [63]
t39	*Daonymphes bisulca*	Nymphidae	Crown Nymphidae	156.0	Makarkin et al. (2013) [64]
t41	*Roesleriana exotica*	Nemopteridae	Crown Nemopteridae	112.0	Martins-Neto and Vulcano (1989) [65]
t42	*Araripeneura gracilis*	Myrmeleontidae	Stem Myrmeleontidae	112.0	Martins-Neto and Vulcano (1989) [65]
t43	*Cratoscalapha electroneura*	Ascalaphidae	Crown Ascalaphidae	112.0	Martins-Neto and Vulcano (1997) [66]

**Table 2:** Divergence time (mean ages and ranges) estimates and branch support values for nodes in Figure 9.

Node	Mean	Inferior 95%	Superior 95%	ESS	PP	Crown Clade
t1	365.60	323.08	407.87	729.30	-	
t2	236.44	178.35	298.66	2526.30	1	Raphidioptera
t3	100.81	58.62	147.11	4757.60	1	Inocellidae
t4	123.72	74.45	173.85	3279.50	1	Raphidiidae
t5	357.55	317.95	398.72	665.80	1	Megaloptera + Neuroptera
t6	295.07	244.99	346.67	1241.40	0.51	Megaloptera
t7	146.42	94.09	213.73	2677.10	1	Sialidae
t8	224.64	173.41	277.62	1362.30	1	Corydalidae
t9	172.69	115.70	227.76	1801.00	0.99	
t10	143.77	99.52	196.41	2175.00	1	
t11	340.63	303.26	379.21	645.90	1	Neuroptera
t12	186.43	144.37	238.87	4926.30	1	Coniopterygidae
t13	315.21	281.93	349.65	612.70	0.88	
t14	286.30	243.32	328.48	1021.00	0.77	Sisyridae + Nevrorthidae
t15	116.65	63.80	174.88	4332.40	1	Nevrorthidae
t16	165.77	103.40	225.00	3088.20	1	Sisyridae
t17	303.89	272.14	336.71	610.00	0.7	
t18	208.93	152.81	266.42	4026.90	1	Osmylidae
t19	286.65	257.58	316.92	602.40	1	
t20	184.70	132.06	234.67	2869.40	1	Dilaridae
t21	269.55	243.48	297.44	605.00	1	
t22	235.64	206.66	263.14	1223.30	1	
t23	218.19	189.95	244.34	1758.50	0.99	
t24	117.49	74.07	161.93	4775.20	1	Berothidae
t25	127.26	90.61	169.15	4104.70	1	Mantispidae
t26	257.94	233.16	284.51	614.80	0.85	
t27	234.12	205.92	262.42	743.80	0.78	
t28	204.80	174.02	235.84	1120.80	0.99	
t29	139.07	101.87	177.78	2996.80	1	Hemerobiidae
t30	121.97	86.50	161.21	3375.90	1	Chrysopidae
t31	207.23	176.14	237.05	890.20	0.99	Ithonidae
t32	179.46	147.53	209.24	970.60	1	
t33	134.03	100.43	170.48	1698.50	1	
t34	148.42	115.70	177.42	1167.70	1	
t35	103.52	71.30	131.58	2017.10	1	
t36	243.91	220.56	269.01	678.10	0.96	
t37	150.38	105.01	199.25	3516.00	1	Psychopsidae
t38	220.46	197.59	244.89	869.30	1	
t39	159.15	135.11	185.78	8146.20	1	Nymphidae
t40	189.59	166.47	213.59	1196.20	1	
t41	123.85	103.30	149.12	6316.90	1	Nemopteridae
t42	154.04	132.89	176.28	1851.30	1	
t43	118.16	100.79	138.64	5110.20	1	Ascalaphidae
t44	118.46	94.31	143.23	3747.90	0.97	Myrmeleontidae

## Data Availability

The genome sequencing data of *I. fulva* (OR506419) and *R. gaoligongensis* (OR506418) that support the findings of this study are openly available in the GenBank of NCBI at https://www.ncbi.nlm.nih.gov (accessed on 6 October 2024). The associated BioProject number is PRJNA1174380. The SRA numbers of *I. fulva* and *R. gaoligongensis* are SRR31035715 and SRR31035716, respectively. The BioSample numbers of *I. fulva* and *R. gaoligongensis* are SAMN44339688 and SAMN44339687, respectively.

## Supplementary Materials

The following supporting information can be downloaded at: https://www.mdpi.com/article/10.3390/insects15120933/s1, Figure S1: The coverage depth of mitochondrial genome of *Ithone fulva* and *Rapisma gaoligongensis*. Figure S2: Phylogenetic tree inferred from Bayesian Inference analysis based on the PCGR_Gb dataset under the CAT-GTR model. Support values on nodes indicate posterior probabilities. Figure S3: Phylogenetic tree inferred from Bayesian Inference analysis based on the 39taxa_PCGR dataset under the CAT-GTR model. Support values on nodes indicate posterior probabilities. Table S1: List of taxonomic groups used for the phylogenetic analyses in this study. Table S2: Organization of mitochondrial genome of *Ithone fulva*. Table S3: Organization of mitochondrial genome of *Rapisma gaoligongensis*. Table S4: Nucleotide composition and skews in the complete mitochondrial genome of Ithonidae. Table S5: Nucleotide composition and skews in the protein-coding genes of the complete mitochondrial genomes of Ithonidae. Table S6: Relative synonymous codon usages (RSCUs) for the protein-coding genes of the mitochondrial genomes of *Ithone fulva*. Table S7: Relative synonymous codon usages (RSCUs) for the protein-coding genes of the mitochondrial genomes of *Rapisma gaoligongensis*. Table S8: Encoded amino acids composition of the protein-coding genes of the complete mitochondrial genomes of Ithonidae. Table S9: Start codons and stop codons of the protein-coding genes of the complete mitochondrial genomes of Ithonidae. Table S10: Nucleotide composition and skews of the rRNAs for the complete mitochondrial genomes of Ithonidae. Table S11: Nucleotide composition and skews of the control regions of the complete mitochondrial genomes of Ithonidae.

## References

[1] Oswald J.D., Machado R.J.P. (2018). Biodiversity of the Neuropterida (Insecta: Neuroptera, Megaloptera, and Raphidioptera). Insect Biodivers..

[2] Winterton S.L., Makarkin V.N. (2010). Phylogeny of moth lacewings and giant lacewings (Neuroptera: Ithonidae, Polystoechotidae) using DNA sequence data, morphology, and fossils. Ann. Entomol. Soc. Am..

[3] Tillyard R.J. (1922). The life-history of the Australian moth-lacewing, *Ithone fusca*, Newman (Order Neuroptera Planipennia). Bull. Entomol. Res..

[4] Grebennikov V.V. (2004). Grub-like larvae of Neuroptera (Insecta): A morphological review of the families Ithonidae and Polystoechotidae and a description of *Oliarces clara*. Eur. J. Entomol..

[5] Li D., Friedrich F., Jandausch K., Pohl H., Liu X., Beutel R.G. (2022). Unearthing underground predators: The head morphology of larvae of the moth lacewing genus *Ithone* Newman (Neuroptera: Ithonidae) and its functional and phylogenetic implications. Syst. Entomol..

[6] Wang Y., Liu X., Winterton S.L., Yan Y., Chang W., Yang D. (2013). Comparative mitogenomic analysis reveals sexual dimorphism in a rare montane lacewing (Insecta: Neuroptera: Ithonidae). PLoS ONE.

[7] Hsiao S.H., Lai B.C. (2022). A new species of the genus *Rapisma* (Insecta: Neuroptera: Ithonidae) from Taiwan. Zool. Stud..

[8] Withycombe C.L. (1925). XV. Some aspects of the biology and morphology of the Neuroptera. with special reference to the immature stages and their possible phylogenetic significance. Trans. R. Entomol. Soc. Lond..

[9] Aspöck U., Plant J.D., Nemeschkal H.L. (2001). Cladistic analysis of Neuroptera and their systematic position within Neuropterida (Insecta: Holometabola: Neuropterida: Neuroptera). Syst. Entomol..

[10] Haring E., Aspöck U. (2004). Phylogeny of the Neuropterida: A first molecular approach. Syst. Entomol..

[11] Beutel R.G., Friedrich F., Aspöck U. (2010). The larval head of Nevrorthidae and the phylogeny of Neuroptera (Insecta). Zool. J. Linn. Soc..

[12] Winterton S.L., Hardy N.B., Wiegmann B.M. (2010). On wings of lace: Phylogeny and Bayesian divergence time estimates of Neuropterida (Insecta) based on morphological and molecular data. Syst. Entomol..

[13] Barnard P.C. (1981). The Rapismatidae (Neuroptera): Montane lacewings of the oriental region. Syst. Entomol..

[14] Penny N.D. (1996). A remarkable new genus and species of Ithonidae from Honduras (Neuroptera). J. Kans. Entomol. Soc..

[15] Liu X. (2017). A review of the montane lacewing genus *Rapisma* McLachlan (Neuroptera, Ithonidae) from China, with description of two new species. Zoosyst. Evol..

[16] Winterton S.L., Lemmon A.R., Gillung J.P., Garzon I.J., Badano D., Bakkes D.K., Breitkreuz L.C.V., Engel M.S., Lemmon E.M., Liu X. (2018). Evolution of lacewings and allied orders using anchored phylogenomics (Neuroptera, Megaloptera, Raphidioptera). Syst. Entomol..

[17] Song N., Li X.-X., Zhai Q., Bozdogan H., Yin X.-M. (2019). The mitochondrial genomes of Neuropteridan insects and implications for the phylogeny of Neuroptera. Genes.

[18] Song N., Lin A., Zhao X. (2018). Insight into higher-level phylogeny of Neuropterida: Evidence from secondary structures of mitochondrial rRNA genes and mitogenomic data. PLoS ONE.

[19] Wang Y., Liu X., Garzón-Orduña I.J., Winterton S.L., Yan Y., Aspöck U., Aspöck H., Yang D. (2017). Mitochondrial phylogenomics illuminates the evolutionary history of Neuropterida. Cladistics.

[20] Machado R.J.P., Gillung J.P., Winterton S.L., Garzón-Orduña I.J., Lemmon A.R., Lemmon E.M., Oswald J.D. (2018). Owlflies are derived antlions: Anchored phylogenomics supports a new phylogeny and classification of Myrmeleontidae (Neuroptera). Syst. Entomol..

[21] Winterton S.L., Gillung J.P., Garzón-Orduña I.J., Badano D., Breitkreuz L.C.V., Duelli P., Engel M.S., Liu X., Machado R.J.P., Mansell M. (2019). Evolution of green lacewings (Neuroptera: Chrysopidae): An anchored phylogenomics approach. Syst. Entomol..

[22] Oswald J.D. Neuropterida Species of the World. https://lacewing.tamu.edu/.

[23] Vasilikopoulos A., Misof B., Meusemann K., Lieberz D., Flouri T., Beutel R.G., Niehuis O., Wappler T., Rust J., Peters R.S. (2020). An integrative phylogenomic approach to elucidate the evolutionary history and divergence times of Neuropterida (Insecta: Holometabola). BMC Evol. Biol..

[24] Tillyard R.J. (1916). Studies in Australian Neuroptera. No. iv. The families Ithonidae, Hemerobiidae, Sisyridae, Berothidae, and the new family Trichomatidae; with a discussion of their characters and relationships, and descriptions of new and little-known genera and species. Proc. Linn. Soc. New South Wales.

[25] Liu X., Li D., Yang Z. (2018). A new species of the montane lacewing genus *Rapisma* McLachlan (Neuroptera, Ithonidae) from China. Zootaxa.

[26] Brown J., Pirrung M., McCue L.A. (2017). FQC Dashboard: Integrates FastQC results into a web-based, interactive, and extensible FASTQ quality control tool. Bioinformatics.

[27] Bolger A.M., Lohse M., Usadel B. (2014). Trimmomatic: A flexible trimmer for Illumina sequence data. Bioinformatics.

[28] Dierckxsens N., Mardulyn P., Smits G. (2017). NOVOPlasty: De novo assembly of organelle genomes from whole genome data. Nucleic Acids Res..

[29] Bernt M., Donath A., Juhling F., Externbrink F., Florentz C., Fritzsch G., Putz J., Middendorf M., Stadler P.F. (2013). MITOS: Improved de novo metazoan mitochondrial genome annotation. Mol. Phylogenet. Evol..

[30] Li H. (2013). Aligning sequence reads, clone sequences and assembly contigs with BWA-MEM. arXiv.

[31] Li H., Handsaker B., Wysoker A., Fennell T., Ruan J., Homer N., Marth G., Abecasis G., Durbin R., 1000 Genome Project Data Processing Subgroup (2009). The sequence alignment/Map format and SAMtools. Bioinformatics.

[32] Greiner S., Lehwark P., Bock R. (2019). OrganellarGenomeDRAW (OGDRAW) version 1.3.1: Expanded toolkit for the graphical visualization of organellar genomes. Nucleic Acids Res..

[33] Kumar S., Stecher G., Li M., Knyaz C., Tamura K. (2018). MEGA X: Molecular evolutionary genetics analysis across computing platforms. Mol. Biol. Evol..

[34] Rozas J., Ferrer-Mata A., Sanchez-DelBarrio J.C., Guirao-Rico S., Librado P., Ramos-Onsins S.E., Sanchez-Gracia A. (2017). DnaSP 6: DNA sequence polymorphism analysis of large data sets. Mol. Biol. Evol..

[35] Bellaousov S., Reuter J.S., Seetin M.G., Mathews D.H. (2013). RNAstructure: Web servers for RNA secondary structure prediction and analysis. Nucleic Acids Res..

[36] Rozewicki J., Li S., Amada K.M., Standley D.M., Katoh K. (2019). MAFFT-DASH: Integrated protein sequence and structural alignment. Nucleic Acids Res..

[37] Kück P., Meusemann K. (2010). FASconCAT: Convenient handling of data matrices. Mol. Phylogenet. Evol..

[38] Castresana J. (2000). Selection of conserved blocks from multiple alignments for their use in phylogenetic analysis. Mol. Biol. Evol..

[39] Talavera G., Vila R. (2011). What is the phylogenetic signal limit from mitogenomes? The reconciliation between mitochondrial and nuclear data in the Insecta class phylogeny. BMC Evol. Biol..

[40] Simon S., Hadrys H. (2013). A comparative analysis of complete mitochondrial genomes among Hexapoda. Mol. Phylogenet. Evol..

[41] Song F., Li H., Jiang P., Zhou X., Liu J., Sun C., Vogler A.P., Cai W. (2016). Capturing the phylogeny of Holometabola with mitochondrial genome data and Bayesian site-heterogeneous mixture models. Genome Biol. Evol..

[42] Cai C., Tihelka E., Giacomelli M., Lawrence J.F., Slipinski A., Kundrata R., Yamamoto S., Thayer M.K., Newton A.F., Leschen R.A.B. (2022). Integrated phylogenomics and fossil data illuminate the evolution of beetles. R. Soc. Open Sci..

[43] Lartillot N., Lepage T., Blanquart S. (2009). PhyloBayes 3: A Bayesian software package for phylogenetic reconstruction and molecular dating. Bioinformatics.

[44] Rambaut A. FigTree. http://tree.bio.ed.ac.uk/software/figtree.

[45] Yang Z. (2007). PAML 4: Phylogenetic analysis by maximum likelihood. Mol. Biol. Evol..

[46] Kendall D.G. (1948). On the generalized ”birth-and-death” process. Ann. Stat..

[47] Nel A., Roques P., Nel P., Prokin A.A., Bourgoin T., Prokop J., Szwedo J., Azar D., Desutter-Grandcolas L., Wappler T. (2013). The earliest known holometabolous insects. Nature.

[48] Rambaut A., Drummond A.J., Xie D., Baele G., Suchard M.A. (2018). Posterior summarization in Bayesian phylogenetics using Tracer 1.7. Syst. Biol..

[49] Carpenter F. (1956). The Baltic amber snake-flies (Neuroptera). Psyche.

[50] Tillyard R.J. (1932). Kansas Permian insects; Part 14, The order Neuroptera. Am. J. Sci..

[51] Prokin A.A., Bashkuev A.S. (2023). The oldest known larvae of Megaloptera (Insecta) from the Triassic of Ukraine. Palaeoentomology.

[52] Meinander M. (1975). Fossil Coniopterygidae. Not. Entomol..

[53] Wichard W., Buder T., Caruso C. (2010). Aquatic lacewings of family Nevrorthidae (Neuroptera) in Baltic amber. Denisia.

[54] Perkovsky E.E., Makarkin V.N. (2015). First confirmation of spongillaflies (Neuroptera: Sisyridae) from the Cretaceous. Cretaceous Res..

[55] Lambkin K. (1988). A re-examination of Lithosmylidia Riek from the Triassic of Queensland with notes on Mesozoic ”osmylid-like” fossil Neuroptera (Insecta: Neuroptera). Mem. Queensl. Mus..

[56] Wang Y., Liu Z., Dong R., Shih C. (2010). A new genus of Protosmylinae from the Middle Jurassic of China (Neuroptera: Osmylidae). Zootaxa.

[57] Engel M.S. (1999). The first fossil of a pleasing lacewing (Neuroptera: Dilaridae). Proc. Entomol. Soc. Wash..

[58] Khramov A., Oyama N., Kenji S., Takahashi H. (2023). Late Triassic lacewings (Insecta: Neuroptera) from Japan. Hist. Biol..

[59] Makarkin V.N., Ohl M. (2015). An important new fossil genus of Berothinae (Neuroptera: Berothidae) from Baltic amber. Zootaxa.

[60] Zheng B.Y., Ren D., Wang Y.J. (2016). Earliest true moth lacewing from the Middle Jurassic of Inner Mongolia, China. Acta Palaeontol. Polonica.

[61] Jepson J.E., Makarkin V.N., Coram R.A. (2012). Lacewings (Insecta: Neuroptera) from the Lower Cretaceous Purbeck Limestone Group of southern England. Cretaceous Res..

[62] Tillyard R.J. (1922). Mesozoic Insects of Queensland. No. 9 Orthoptera, and additions to the Protorthoptera, Odonata, Hemiptera and Planipennia. Proc. Linn. Soc. New South Wales.

[63] Ren D., Engel M.S. (2007). A split-footed lacewing and two epiosmylines from the Jurassic of China (Neuroptera). Ann. Zool..

[64] Makarkin V.N., Yang Q., Shi C.F., Ren D. (2013). The presence of the recurrent veinlet in the Middle Jurassic Nymphidae (Neuroptera): A unique character condition in Myrmeleontoidea. Zookeys.

[65] Martins-Neto R.G., Vulcano M.A. (1989). Neurópteros (Insecta, Planipennia) da Formação Santana (Cretáceo Inferior), Bacia do Araripe, nordeste do Brasil. II. Superfamília Myrmeleontoidea. Rev. Bras. Entomol..

[66] Martins-Neto R.G., Vulcano M.A. (1997). Neurópteros (Insecta, Planipennia) da Formação Santana (Cretáceo Inferior), Bacia do Araripe, Nordeste do Brasil. VIII—Descrição de novos taxa de Myrmeleontidae, Ascalaphidae Nemopteridadae. Rev. Univ. Guarulhos Ciências Biológicas E Saúde.

[67] Beckenbach A.T., Stewart J.B. (2009). Insect mitochondrial genomics 3: The complete mitochondrial genome sequences of representatives from two neuropteroid orders: A dobsonfly (order Megaloptera) and a giant lacewing and an owlfly (order Neuroptera). Genome.

[68] Tian S., Jiang Y., Lai Y., Wang S., Liu X., Wang Y. (2023). New mitogenomes of the green lacewing tribe Ankylopterygini (Neuroptera: Chrysopidae: Chrysopinae) and phylogenetic implications of Chrysopidae. Insects.

[69] Xu H., Wu Y., Wang Y., Liu Z. (2020). Comparative analysis of five mitogenomes of Osmylinae (Neuroptera: Osmylidae) and their phylogenetic implications. Int. J. Biol. Macromol..

[70] Liu W., Wang C., Wang J., Tang Y., Pei W., Ge X., Yan C. (2024). Phylogenetic and comparative analysis of *Cryptochironomus*, *Demicryptochironomus* and *Harnischia* inferred from mitogenomes (Diptera: Chironomidae). Insects.

[71] Ji L., Jia Z., Bai X. (2024). Comparative analysis of the mitochondrial genomes of three species of *Yangiella* (Hemiptera: Aradidae) and the phylogenetic implications of Aradidae. Insects.

[72] Hurst L.D. (2009). Genetics and the understanding of selection. Nat. Rev. Genet..

[73] Hurst L.D. (2002). The Ka/Ks ratio: Diagnosing the form of sequence evolution. Trends Genet..

[74] Cannone J.J., Subramanian S., Schnare M.N., Collett J.R., D’Souza L.M., Du Y., Feng B., Lin N., Madabusi L.V., Müller K.M. (2002). The Comparative RNA Web (CRW) Site: An online database of comparative sequence and structure information for ribosomal, intron, and other RNAs. BMC Bioinform..

[75] Zhao Y., Zhang H., Zhang Y. (2017). Complete mitochondrial genome of *Neochauliodes parasparsus* (Megaloptera: Corydalidae) with phylogenetic consideration. Biochem. Syst. Ecol..

[76] Zhao J., Li H., Winterton S.L., Liu Z. (2013). Ancestral gene organization in the mitochondrial genome of *Thyridosmylus langii* (McLachlan, 1870) (Neuroptera: Osmylidae) and implications for lacewing evolution. PLoS ONE.

[77] Zhang L., Yang J. (2017). The mitochondrial genome of *Gatzara jezoensis* (Neuroptera: Myrmeleontidae) and phylogenetic analysis of Neuroptera. Biochem. Syst. Ecol..

[78] Zhang C., Liu X., Liu C., Luo X. (2020). Characterization of the complete mitochondrial genome of *Acanthacorydalis fruhstorferi* van der Weele (Megaloptera: Corydalidae). J. Kans. Entomol. Soc..

[79] Zhan Q., Gai Y., Zhao Y. (2024). Characterization of the complete mitochondrial genome of the *Libelloides sibiricus* (Neuroptera, Ascalaphidae). Mitochondrial. DNA Part B.

[80] Beutel R.G., Friedrich F., Hoernschemeyer T., Pohl H., Huenefeld F., Beckmann F., Meier R., Misof B., Whiting M.F., Vilhelmsen L. (2011). Morphological and molecular evidence converge upon a robust phylogeny of the megadiverse Holometabola. Cladistics.

[81] Aspöck U., Aspöck H. (2008). Phylogenetic relevance of the genital sclerites of Neuropterida (Insecta: Holometabola). Syst. Entomol..

[82] Zhao C., Liu X., Yang D. (2014). Wing base structural data support the sister relationship of Megaloptera and Neuroptera (Insecta: Neuropterida). PLoS ONE.

[83] Zhao C., Huang M., Yang D., Liu X. (2024). Comparative morphology of the wing base structure illuminates higher-level phylogeny of Holometabola. Insects.

[84] Misof B., Liu S., Meusemann K., Peters R.S., Donath A., Mayer C., Frandsen P.B., Ware J., Flouri T., Beutel R.G. (2014). Phylogenomics resolves the timing and pattern of insect evolution. Science.

[85] Wang Y., Zhou X., Wang L., Liu X., Yang D., Rokas A. (2019). Gene selection and evolutionary modeling affect phylogenomic inference of Neuropterida based on transcriptome data. Int. J. Mol. Sci..

[86] Amorim A., Fernandes T., Taveira N. (2019). Mitochondrial DNA in human identification: A review. PeerJ.

[87] Ballard J.W., Whitlock M.C. (2004). The incomplete natural history of mitochondria. Mol. Ecol..

[88] Simon C., Buckley T.R., Frati F., Stewart J.B., Beckenbach A.T. (2006). Incorporating molecular evolution into phylogenetic analysis, and a new compilation of conserved polymerase chain reaction primers for animal mitochondrial DNA. Annu. Rev. Ecol. Evol. Syst..

[89] Hurley I.A., Mueller R.L., Dunn K.A., Schmidt E.J., Friedman M., Ho R.K., Prince V.E., Yang Z., Thomas M.G., Coates M.I. (2007). A new time-scale for ray-finned fish evolution. Proc. Biol. Sci..

[90] Duchene S., Lanfear R., Ho S.Y. (2014). The impact of calibration and clock-model choice on molecular estimates of divergence times. Mol. Phylogenet. Evol..

[91] Inoue J., Donoghue P.C., Yang Z. (2010). The impact of the representation of fossil calibrations on Bayesian estimation of species divergence times. Syst. Biol..

[92] Ho S.Y.W., Phillips M.J. (2009). Accounting for calibration uncertainty in phylogenetic estimation of evolutionary divergence times. Syst. Biol..

