# Supplementary material for: New Mitochondrial Genomes of Ithonidae (Neuroptera) and Higher Phylogenetic Implications
Source: Insects. 2024 Nov 27;15(12):933. doi: 10.3390/insects15120933 (PMC11677771; doi:10.3390/insects15120933)
Supplement: Supplementary file 1 [file insects-15-00933-s001.zip › Table S1-11.pdf]

Table S1. List of taxonomic groups used for the phylogenetic analyses in this study.

Family	Species	Accession number	Length
**Outgroups**			
Inocelliidae	*Amurinocellia sinica*	NC_070254	15,916 bp
Inocelliidae	*Negha inflata*	NC_070258	15,952 bp
Raphidiidae	*Mongoloraphidia harmandi*	NC_013251	16,006 bp
Raphidiidae	*Agulla arizonica*	NC_070270	15,639 bp
Sialidae	*Stenosialis australiensis*	KT425084	14,018 bp
Sialidae	*Sialis melania*	MW642263	15,587 bp
Corydalidae	*Protohermes concolorus*	NC_011524	15,851 bp
Corydalidae	*Neochauliodes punctatolosus*	NC_018772	15,734 bp
Corydalidae	*Acanthacorydalis orientalis*	NC_023462	15,753 bp
Corydalidae	*Dysmicohermes ingens*	NC_024657	16,271 bp
**Ingroups**			
Ascalaphidae	*Ogcogaster segmentator*	NC_068272	15,916 bp
Ascalaphidae	*Suhpalacsa longialata*	NC_039948	15,911 bp
Berothidae	*Isoscelipteron pectinatum*	MZ465548	15,840 bp
Berothidae	*Stenobiella* sp.	KT425081	14,374 bp
Chrysopidae	*Chrysopa formosa*	NC_084372	16,104 bp
Chrysopidae	*Nothochrysa sinica*	NC_060341	16,166 bp
Coniopterygidae	*Semidalis macleodi*	NC_061560	16,571 bp
Coniopterygidae	*Conwentzia sinica*	NC_057219	15,153 bp
Dilaridae	*Nallachius americanus*	KT425071	12,810 bp
Dilaridae	*Dilar* sp.	KT425073	14,173 bp
Hemerobiidae	*Micromus paganus*	NC_060415	16,607 bp
Hemerobiidae	*Hemerobius spodipennis*	NC_051001	16,343 bp
Ithonidae	*Oliarces clara*	KT425090	16,101 bp
Ithonidae	*Polystoechotes punctata*	NC_011278	16,036 bp
Ithonidae	*Fontecilla graphicus*	KT425072	14,017 bp

Ithonidae	*Rapisma zayuanum*	NC_023363	15,984 bp
Ithonidae	*Ithone fulva*	OR506419	This study
Ithonidae	*Rapisma gaoligongensis*	OR506418	This study
Mantispidae	*Mantispa japonica*	NC_039771	16,106 bp
Mantispidae	*Eumantispa harmandi*	NC_039772	15,741 bp
Myrmeleontidae	*Yunleon longicorpus*	NC_085285	16,810 bp
Myrmeleontidae	*Myrmeleon formicarius*	NC_047285	16,179 bp
Nemopteridae	*Nemopistha sinica*	NC_086508	15,839 bp
Nemopteridae	*Nemoptera coa*	NC_086509	15,839 bp
Nevrorthidae	*Nipponeurorthus fuscinervis*	KT425076	14,447 bp
Nevrorthidae	*Nevrorthus apatelios*	KT425074	14,581 bp
Nymphidae	*Nymphes myrmeleonoides*	NC_024825	15,712 bp
Nymphidae	*Myiodactylus osmyloides*	KT425089	14,725 bp
Osmylidae	*Gryposmylus pennyi*	NC_053271	16,255 bp
Osmylidae	*Osmylus fulvicephalus*	NC_050653	16,280 bp
Psychopsidae	*Balmes birmanus*	KT425083	16,192 bp
Psychopsidae	*Psychopsis coelivaga*	KT425082	14,713 bp
Sisyridae	*Sisyra aurorae*	MZ159968	15,634 bp
Sisyridae	*Climacia areolaris*	KT425088	15,382 bp
Rhachiberothidae	*Mucroberotha vesicaria*	KT425092	12,047 bp

Table S2. Organization of mitochondrial genome of *Ithone fulva*.

Gene	Direction	Location		Size	Anticodon	Codon		InteNgenic
		Start	End			Start	Stop	nucleotides
tRNA^{Ile}	H	1	66	66	GAT			0
tRNA^{Gln}	L	229	297	69	TTG			162
tRNA^{Met}	H	301	370	70	CAT			3
ND2	H	371	1382	1012		ATT	T-tRNA	0
tRNA^{Cys}	L	1383	1447	65	GCA			0
tRNA^{Trp}	H	1448	1513	66	TCA			0
tRNA^{Tyr}	L	1515	1581	67	GTA			1
COX1	H	1620	3117	1498		ATT	T-tRNA	38
tRNA$^{Leu(UUR)}$	H	3118	3181	64	TAA			0
COX2	H	3184	3868	685		ATG	T-tRNA	2
tRNA^{Lys}	H	3869	3939	71	CTT			0
tRNA^{Asp}	H	3940	4007	68	GTC			0
ATP8	H	4008	4166	159		ATT	TAA	0
ATP6	H	4160	4837	678		ATG	TAA	-7
COX3	H	4837	5624	788		ATG	TA-tRNA	-1
tRNA^{Gly}	H	5625	5689	65	TCC			0
ND3	H	5690	6038	349		ATA	T-tRNA	0
tRNA^{Ala}	H	6039	6104	66	TGC			0
tRNA^{Arg}	H	6104	6167	64	TCG			-1
tRNA^{Asn}	H	6167	6232	66	GTT			-1
tRNA$^{Ser(AGN)}$	H	6233	6300	68	GCT			0
tRNA^{Glu}	H	6301	6365	65	TTC			0
tRNA^{Phe}	L	6365	6429	65	GAA			-1
ND5	L	6430	8158	1729		ATT	T-tRNA	0
tRNA^{His}	L	8159	8225	67	GTG			0
ND4	L	8226	9561	1336		ATG	T-tRNA	0

ND4L	L	9555	9851	297		ATG	TAA	-7
tRNA^{Thr}	H	9868	9932	65	TGT			16
tRNA^{Pro}	L	9933	9999	67	TGG			0
ND6	H	10001	10519	519		ATT	TAA	1
CytB	H	10519	11653	1135		ATG	T-tRNA	-1
tRNA$^{Ser(UCN)}$	H	11654	11720	67	TGA			0
ND1	L	11742	12671	930		ATA	TAG	21
tRNA$^{Leu(CUN)}$	L	12694	12757	64	TAG			22
rrnL	L	12758	14078	1321				0
tRNA^{Val}	L	14079	14151	73	TAC			0
rrnS	L	14152	14936	785				0
Control region		14937	16041	1105				0

Table S3. Organization of mitochondrial genome of *Rapisma gaoligongensis*.

Gene	Direction	Location		Size	Anticodon	Codon		InteNgenic
		Start	End			Start	Stop	nucleotides
tRNA^{Ile}	H	1	63	63	GAT			0
tRNA^{Gln}	L	121	189	69	TTG			57
tRNA^{Met}	H	194	265	72	CAT			4
ND2	H	266	1280	1015		ATC	T-tRNA	0
tRNA^{Cys}	L	1281	1346	66	GCA			0
tRNA^{Trp}	H	1369	1434	66	TCA			22
tRNA^{Tyr}	L	1437	1503	67	GTA			2
COX1	H	1508	3046	1539		ATA	TAA	4
tRNA$^{Leu(UUR)}$	H	3194	3259	66	TAA			147
COX2	H	3262	3946	685		ATG	T-tRNA	2
tRNA^{Lys}	H	3947	4018	72	CTT			0
tRNA^{Asp}	H	4018	4084	67	GTC			-1
ATP8	H	4085	4240	156		ATT	TAA	0
ATP6	H	4234	4908	675		ATG	TAA	-7
COX3	H	4913	5700	788		ATG	TA-tRNA	4
tRNA^{Gly}	H	5701	5764	64	TCC			0
ND3	H	5765	6118	354		ATA	TAA	0
tRNA^{Ala}	H	6123	6186	64	TGC			4
tRNA^{Arg}	H	6186	6250	65	TCG			-1
tRNA^{Asn}	H	6251	6315	65	GTT			0
tRNA$^{Ser(AGN)}$	H	6316	6382	67	GCT			0
tRNA^{Glu}	H	6390	6455	66	TTC			7
tRNA^{Phe}	L	6454	6523	70	GAA			-2
ND5	L	6524	8243	1720		ATT	T-tRNA	0
tRNA^{His}	L	8244	8307	64	GTG			0
ND4	L	8308	9640	1333		ATG	T-tRNA	0

ND4L	L	9634	9921	288		ATG	TAA	-7
tRNA^{Thr}	H	9924	9988	65	TGT			2
tRNA^{Pro}	L	9989	10056	68	TGG			0
ND6	H	10058	10564	507		ATT	TAA	1
CytB	H	10564	11698	1135		ATG	T-tRNA	-1
tRNA$^{Ser(UCN)}$	H	11699	11765	67	TGA			0
ND1	L	11789	12736	948		TTG	TAG	23
tRNA$^{Leu(CUN)}$	L	12738	12800	63	TAG			1
rrnL	L	12801	14122	1322				0
tRNA^{Val}	L	14123	14192	70	TAC			0
rrnS	L	14193	14987	795				0
Control region		14988	16156	1169				0

Table S4. Nucleotide composition and skews in the complete mitochondrial genome of Ithonidae.

Species	T(U)	C	A	G	Total	A+T%	AT-Skew	GC-Skew
Ithone fulva	40.34	12.27	38.45	8.94	16041	78.79	-0.02	-0.16
Oliarces clara	42.03	12.40	37.37	8.20	16101	79.41	-0.06	-0.20
Polystoechotes punctata	40.61	12.22	38.34	8.82	16036	78.96	-0.03	-0.16
Rapisma gaoligongensis	42.65	10.31	40.18	6.86	16156	82.83	-0.03	-0.20
Rapisma zayuanum	43.28	11.20	37.81	7.71	15984	81.09	-0.07	-0.18

Table S5. Nucleotide composition and skews in the protein-coding genes of the complete mitochondrial genomes of Ithonidae.

Species	T(U)	C	A	G	Total	A+T%	AT-Skew	GC-Skew
Ithone fulva	39.70	12.93	37.62	9.74	11115	77.33	-0.03	-0.14
Oliarces clara	41.03	13.27	36.62	9.08	11173	77.64	-0.06	-0.19
Polystoechotes punctata	39.89	12.94	37.33	9.83	11167	77.23	-0.03	-0.14
Rapisma gaoligongensis	42.08	11.09	39.18	7.65	11143	81.26	-0.04	-0.18
Rapisma zayuanum	42.52	11.98	36.78	8.72	11164	79.30	-0.07	-0.16

Table S6. Relative synonymous codon usages (RSCUs) for the protein-coding genes of the mitochondrial genomes of *Ithone fulva*.

Codon	Count	RSCU	Codon	Count	RSCU
UUU(F)	308	1.86	UCU(S)	113	2.14
UUC(F)	23	0.14	UCC(S)	8	0.15
UUA(L)	525	1.96	UCA(S)	88	1.67
UUG(L)	12	0.04	UCG(S)	2	0.04
UAU(Y)	162	1.83	UGU(C)	30	1.76
UAC(Y)	15	0.17	UGC(C)	4	0.24
UAA(*)	4	1.60	UGA(W)	91	1.90
UAG(*)	1	0.40	UGG(W)	5	0.10
CUU(L)	45	2.34	CCU(P)	79	2.39
CUC(L)	1	0.05	CCC(P)	14	0.42
CUA(L)	30	1.56	CCA(P)	38	1.15
CUG(L)	1	0.05	CCG(P)	1	0.03
CAU(H)	67	1.81	CGU(R)	15	1.05
CAC(H)	7	0.19	CGC(R)	0	0.00
CAA(Q)	66	2.00	CGA(R)	40	2.81
CAG(Q)	5	0.00	CGG(R)	2	0.14
AUU(I)	382	1.91	ACU(T)	92	2.13
AUC(I)	17	0.09	ACC(T)	12	0.28
AUA(M)	212	1.90	ACA(T)	69	1.60
AUG(M)	11	0.10	ACG(T)	0	0.00
AAU(N)	192	1.87	AGU(S)	46	1.48
AAC(N)	13	0.13	AGC(S)	7	0.23
AAA(K)	80	1.76	AGA(S)	71	2.29
AAG(K)	11	0.24	AGG(S)	0	0.00
GUU(V)	77	1.88	GCU(A)	100	2.45
GUC(V)	1	0.02	GCC(A)	10	0.25

GUA(V)	81	1.98	GCA(A)	51	1.25
GUG(V)	5	0.12	GCG(A)	2	0.05
GAU(D)	65	1.81	GGU(G)	70	1.32
GAC(D)	7	0.19	GGC(G)	6	0.11
GAA(E)	73	1.97	GGA(G)	118	2.23
GAG(E)	1	0.03	GGG(G)	18	0.34

*：Stop condon

Table S7. Relative synonymous codon usages (RSCUs) for the protein-coding genes of the mitochondrial genomes of *Rapisma gaoligongensis*.

Codon	Count	RSCU	Codon	Count	RSCU
UUU(F)	360	1.95	UCU(S)	123	2.23
UUC(F)	9	0.05	UCC(S)	4	0.07
UUA(L)	538	1.95	UCA(S)	92	1.67
UUG(L)	15	0.05	UCG(S)	2	0.04
UAU(Y)	186	1.96	UGU(C)	33	2.00
UAC(Y)	4	0.04	UGC(C)	0	0.00
UAA(*)	6	1.71	UGA(W)	96	1.94
UAG(*)	1	0.29	UGG(W)	3	0.06
CUU(L)	27	2.40	CCU(P)	71	2.45
CUC(L)	3	0.27	CCC(P)	7	0.24
CUA(L)	15	1.33	CCA(P)	37	1.28
CUG(L)	0	0.00	CCG(P)	1	0.03
CAU(H)	67	1.89	CGU(R)	21	1.58
CAC(H)	4	0.11	CGC(R)	0	0.00
CAA(Q)	64	2.00	CGA(R)	30	2.26
CAG(Q)	3	0.00	CGG(R)	2	0.15
AUU(I)	461	1.94	ACU(T)	95	2.35
AUC(I)	14	0.06	ACC(T)	2	0.05
AUA(M)	271	1.94	ACA(T)	64	1.58
AUG(M)	9	0.06	ACG(T)	1	0.02
AAU(N)	226	1.97	AGU(S)	27	0.92
AAC(N)	4	0.03	AGC(S)	4	0.14
AAA(K)	94	1.92	AGA(S)	86	2.92
AAG(K)	4	0.08	AGG(S)	1	0.03
GUU(V)	43	1.79	GCU(A)	66	2.59
GUC(V)	1	0.04	GCC(A)	2	0.08

GUA(V)	51	2.13	GCA(A)	33	1.29
GUG(V)	1	0.04	GCG(A)	1	0.04
GAU(D)	66	1.97	GGU(G)	39	0.84
GAC(D)	1	0.03	GGC(G)	3	0.06
GAA(E)	71	1.89	GGA(G)	131	2.83
GAG(E)	4	0.11	GGG(G)	12	0.26

*：Stop condon

Table S8. Encoded amino acids composition of the protein-coding genes of the complete mitochondrial genomes of Ithonidae.

Codon	*Ithone fulva*	*Oliarces clara*	*Polystoechotes punctata*	*Rapisma gaoligongensis*	*Rapisma zayuanum*
Ala	4.50%	4.12%	4.52%	2.75%	3.58%
Arg	1.57%	1.56%	1.56%	1.43%	1.48%
Asn	5.66%	5.71%	5.55%	6.19%	6.11%
Asp	0.00%	1.80%	1.78%	1.80%	1.83%
Cys	0.94%	1.00%	1.05%	0.89%	0.94%
Glu	2.04%	2.21%	2.29%	2.02%	2.05%
Gln	1.96%	1.80%	1.83%	1.80%	1.72%
Gly	5.85%	5.39%	5.79%	4.98%	5.28%
His	2.04%	2.05%	2.13%	1.91%	1.97%
Ile	11.01%	10.88%	10.31%	12.79%	12.17%
Leu	16.94%	15.99%	16.24%	16.10%	16.40%
Lys	2.51%	2.50%	2.15%	2.64%	2.67%
Met	6.15%	5.82%	5.22%	7.54%	6.25%
Phe	9.13%	10.29%	9.24%	9.94%	9.77%
Pro	3.64%	3.53%	3.55%	3.12%	3.34%
Ser	9.24%	9.05%	9.07%	9.13%	9.24%
Thr	4.77%	5.06%	5.25%	4.36%	4.39%
Trp	2.65%	2.61%	2.72%	2.67%	2.67%
Tyr	4.88%	4.52%	4.74%	5.12%	4.82%
Val	4.52%	4.12%	4.95%	2.58%	3.26%

Table S9. Start codons and stop codons of the protein-coding genes of the complete mitochondrial genomes of Ithonidae.

Species	*ATP6*	*ATP8*	*COX1*	*COX2*	*COX3*	*CYTB*	*ND1*	*ND2*	*ND3*	*ND4*	*ND4L*	*ND5*	*ND6*
**Start codon**													
Ithone fulva	ATG	ATT	ATT	ATG	ATG	ATG	ATA	ATT	ATA	ATG	ATG	ATT	ATT
Oliarces clara	ATG	ATT	CGA	ATG	ATG	ATG	TTG	ATT	ATT	ATG	ATG	ATT	ATC
Polystoechotes punctata	ATG	ATT	TCG	ATG	ATG	ATG	ATG	ATT	ATT	ATG	ATG	ATA	ATC
Rapisma gaoligongensis	ATG	ATT	ATA	ATG	ATG	ATG	TTG	ATC	ATA	ATG	ATG	ATT	ATT
Rapisma zayuanum	ATG	ATT	ATT	ATG	ATG	ATG	TTG	ATT	ATT	ATG	ATA	ATA	ATA
**Stop codon**													
Ithone fulva	TAA	TAA	T-tRNA	T-tRNA	TA-tRNA	T-tRNA	TAG	T-tRNA	T-tRNA	T-tRNA	TAA	T-tRNA	TAA
Oliarces clara	TAA	TAA	T-tRNA	T-tRNA	TAA	TAA	TAG	TAA	TAA	T-tRNA	TAA	T-tRNA	TAA
Polystoechotes punctata	TAA	TAA	T-tRNA	T-tRNA	TAA	TAA	TAG	TAA	TAA	T-tRNA	TAA	T-tRNA	TAA
Rapisma gaoligongensis	TAA	TAA	TAA	T-tRNA	TA-tRNA	T-tRNA	TAG	T-tRNA	TAA	T-tRNA	TAA	T-tRNA	TAA
Rapisma zayuanum	TAA	TAA	T-tRNA	T-tRNA	TAA	TAA	TAG	TAA	TAA	T-tRNA	TAA	T-tRNA	TAA

Table S10. Nucleotide composition and skews of the rRNAs for the complete mitochondrial genomes of Ithonidae.

Species	T(U)	C	A	G	Total	A+T%	AT-Skew	GC-Skew
rrnL								
Ithone fulva	40.42	6.51	41.56	11.51	1321	81.98	0.01	0.28
Oliarces clara	38.58	5.95	43.33	12.13	1327	81.91	0.06	0.34
Polystoechotes punctata	38.67	6.82	42.84	11.68	1319	81.50	0.05	0.26
Rapisma gaoligongensis	41.98	5.82	41.60	10.59	1322	83.59	0.00	0.29
Rapisma zayuanum	38.45	5.93	44.07	11.55	1316	82.52	0.07	0.32
rrnS								
Ithone fulva	38.85	7.77	41.02	12.36	785	79.87	0.03	0.23
Oliarces clara	35.39	7.18	44.96	12.47	794	80.35	0.12	0.27
Polystoechotes punctata	38.14	7.14	41.84	12.88	784	79.97	0.05	0.29
Rapisma gaoligongensis	41.76	5.16	43.52	9.56	795	85.28	0.02	0.30
Rapisma zayuanum	38.22	5.92	44.92	10.94	777	83.14	0.08	0.30

Table S11. Nucleotide composition and skews of the control regions of the complete mitochondrial genomes of Ithonidae.

Species	T(U)	C	A	G	Total	A+T%	AT-Skew	GC-Skew
Ithone fulva	47.24	7.87	39.19	5.70	1105	86.43	-0.09	-0.16
Oliarces clara	51.08	4.85	41.82	2.25	1155	92.90	-0.10	-0.37
Polystoechotes punctata	46.22	5.43	46.84	1.51	1123	93.05	0.01	-0.56
Rapisma gaoligongensis	48.33	4.62	45.34	1.71	1169	93.67	-0.03	-0.46
Rapisma zayuanum	50.80	4.02	44.09	1.09	1193	94.89	-0.07	-0.57
