# Supplementary figures and images for: New Mitochondrial Genomes of Ithonidae (Neuroptera) and Higher Phylogenetic Implications
Source: Insects. 2024 Nov 27;15(12):933. doi: 10.3390/insects15120933 (PMC11677771; doi:10.3390/insects15120933)

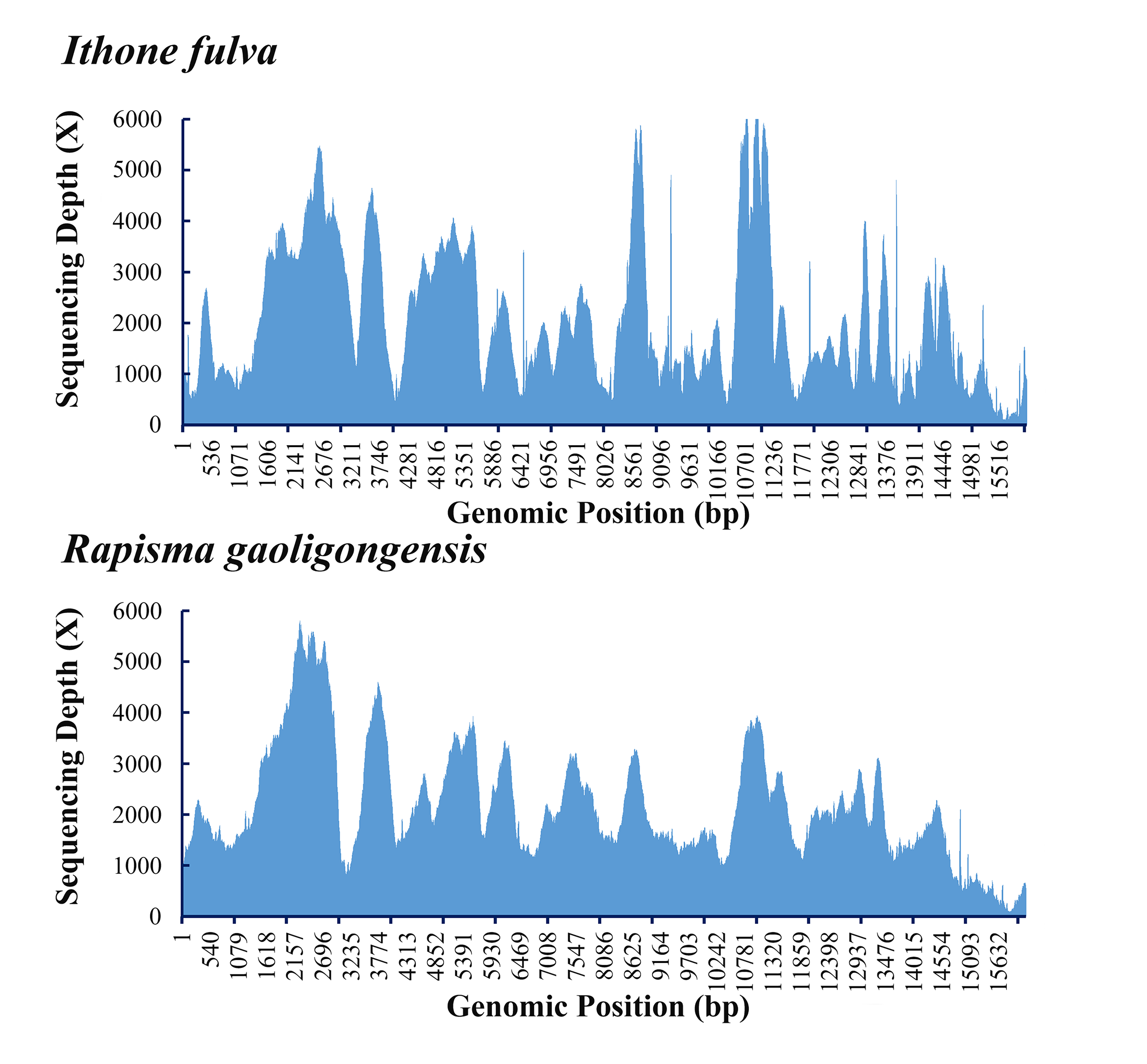

Supplement: Supplementary file 1 [file insects-15-00933-s001.zip › Figure S1.tif]

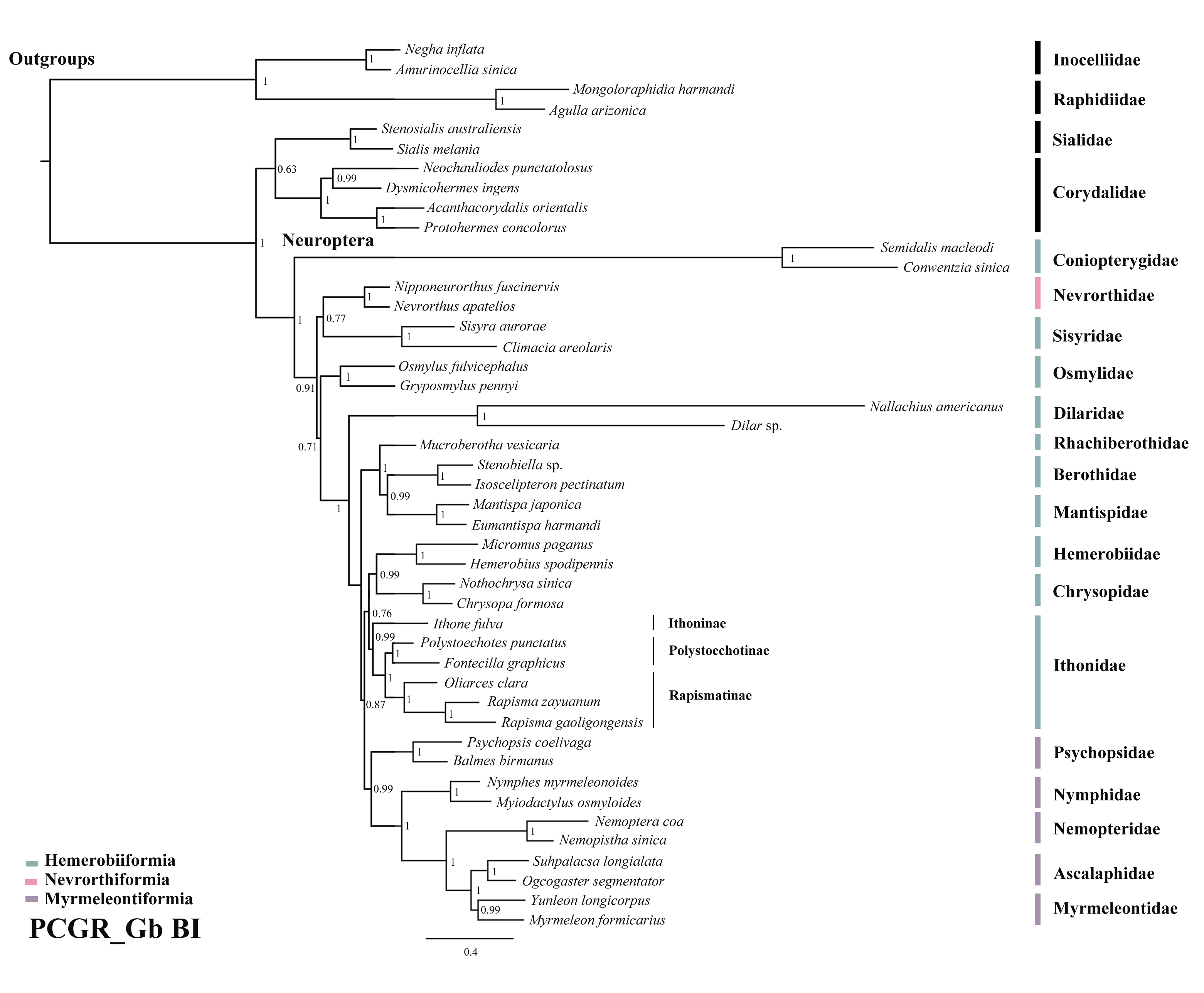

Supplement: Supplementary file 1 [file insects-15-00933-s001.zip › Figure S2.tif]

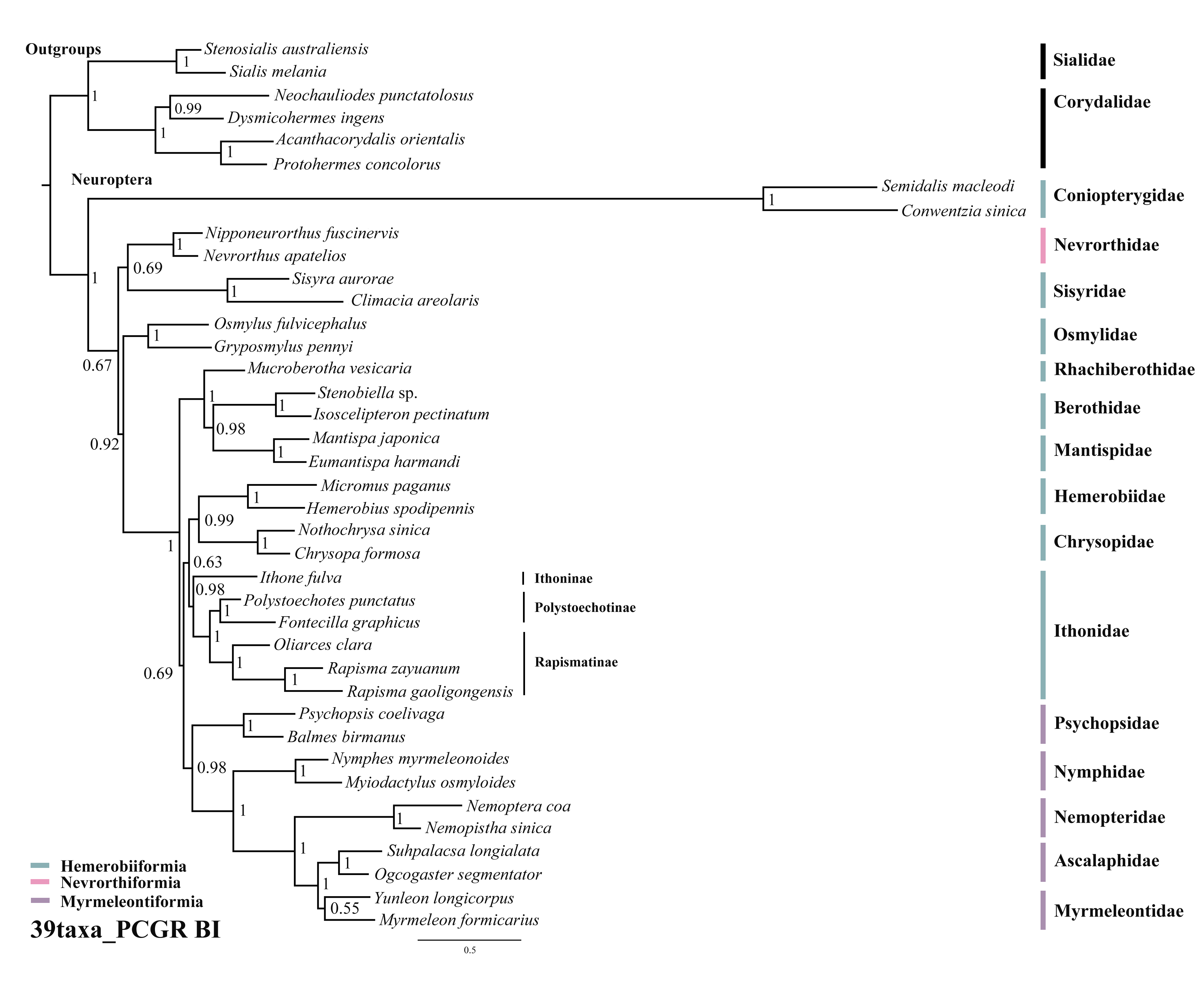

Supplement: Supplementary file 1 [file insects-15-00933-s001.zip › Figure S3.tif]
